# EEG Oscillatory Networks in Peri-Ictal Period of Absence Epilepsy

**DOI:** 10.3389/fneur.2022.825225

**Published:** 2022-04-25

**Authors:** Zhiye Li, Jialing Huang, Wei Wei, Sili Jiang, Hong Liu, Hua Luo, Jianghai Ruan

**Affiliations:** ^1^Department of Neurology, The Affiliated Hospital of Southwest Medical University, Luzhou, China; ^2^Laboratory of Neurological Diseases and Brain Function, Luzhou, China; ^3^Department of Neurology, Suining Central Hospital, Suining, China

**Keywords:** absence epilepsy, seizures, independent component analysis, electroencephalography, brain networks

## Abstract

**Objective:**

To investigate the dynamical brain network changes before and after an absence seizure episode in absence epilepsy (AE).

**Methods:**

21 AE patients with a current high frequency of seizures and 21 sex- and age-matched health control (HC) who reported no history of neurological or psychiatric disorders and visited the hospital for routine physical examinations were included. Each included subject underwent a 2-h and 19-channel video EEG examination. For AE patients, five epochs of 10-s EEG data in inter-ictal, pre-ictal, and post-ictal states were collected. For the HC group, five 10-s resting-state EEG epochs were extracted. Functional independent components analysis (ICA) was carried out using the LORETA KEY tool.

**Results:**

Compared with the resting-state EEG data of the HC group, the EEG data from AE patients during inter-ictal periods showed decreased alpha oscillations in regions involving the superior frontal gyrus (SFG) (BA11). From inter-ictal to pre-ictal, SFG (BA10) showed maximum decreased delta oscillations. Additionally, from pre-ictal to post-ictal, superior temporal gyrus (STG) (BA 22) presented maximum increased neural activity in the alpha band. Moreover, compared with inter-ictal EEG, post-ictal EEG showed significantly decreased theta activity in SFG (BA8).

**Conclusion:**

The changes in SFG alpha oscillations are the key brain network differences between inter-ictal EEG of AE patients and resting-state EEG of HCs. The brain networks of EEG oscillatory during peri-ictal episodes are mainly involving SFG and STG. Our study suggests that altered EEG brain networks dynamics exist between inter-ictal EEG of AE patients and resting-state EEG of HCs and between pre- and post-ictal EEG in AE patients.

## Introduction

Childhood absence epilepsy (AE) accounts for 10–17% of all cases of childhood-onset epilepsy, making it the most common type of epilepsy in childhood (1, 2). The clinical syndrome is characterized by frequent but brief staring spells. Generally, typical 3–4 Hz generalized spike-wave discharges (GSWDs) will be found in the ictal period (3). AE is easy to be diagnosed, can be effectively treated. However, some studies have confirmed that it has a significant impact on children's lives, who has a higher rate of impaired behavior, emotions, cognition, and language and low intervention rate. These problems can persist into adulthood with a lower level of employment and more social isolation (4, 5).

Over the past decades, advances in neuroimaging and EEG techniques have led to a better understanding of the brain networks involved in AE. Some studies have found that the characteristic generalized spike-and-wave discharges are associated with “activations” and “deactivations” within a network of cortical or subcortical brain regions (6, 7). Interestingly, the changes of functional connectivity (FC) of AE patients in the pre-treatment ictal state may indicate the response to antiepileptic treatment (8). In addition, our previous study has recognized that the EEG microstates of AE before and after absence seizures are characterized by a “slowdown” in transitions between microstates (9). These studies are meaningful for interpreting the different patterns of brain activity that are changing during the peri-ictal period of AE. However, the detailed evolutions of brain networks during the peri-ictal time in patients with AE remain to be solved. Thus, we continue to explore the pattern of brain networks in inter-ictal, pre-ictal and post-ictal periods.

A widely used brain network analysis method, independent component analysis (ICA) is a classical electroencephalogram (EEG) signal analysis technique, which separates scalp electrical signals into their additive independent or source components (10, 11). All components activated in the results are statistically independent of each other to identify independent spatial brain networks and their interactions in frequency bands (12), which means that different networks do not activate together. This method, using EEG data, shows a high time resolution (13), and a relatively good spatial resolution. Therefore, we planned to use the EEG ICA method to explore the changes of brain networks during peri-ictal periods in patients AE.

In the present study, we hypothesized that the occurrence and termination of absence episodes are accompanied by the change of brain functional networks. We aimed to investigate whether and how brain functional networks changed under three conditions, including inter-ictal, pre-ictal, and post-ictal states, in AE patients.

## Patients and Methods

### Participants

A total of 21 patients with typical childhood AE were recruited from January 2016 until January 2019 in the neurology department of the Affiliated Hospital of Southwest Medical University. Inclusion criteria: (a) clinical diagnosis of childhood AE was established according to the International League against Epilepsy (ILAE) for a revised classification of epilepsy and epileptic syndromes (14); (b) EEG showed typical bilateral, synchronous ca. 3 Hz GSWDs during seizure episodes, with a high frequency of absence seizures (more than 5 seizures within the 2-h video EEG examination); (c) no anatomical abnormality in routine structural Magnetic Resonance Imaging (MRI) examinations; (d) no additional seizure types, such as systemic tonic-clonic or myoclonic seizures. Exclusion criteria: (a) Previous history of other major neuropsychiatric disorders; (b) Large interference and poor effect of EEG data; All included subjects were right-handed. In addition, 21 age- and sex-matched healthy controls without epilepsy and other neurological diseases were included. This data sample has been partly reported by our group in a previous study (9). Written informed consent was obtained from all participants. The study was approved by the medical ethics committee of the Affiliated Hospital of Southwest Medical University.

### Preparation Before EEG Collection

Subjects washed and dried their hair before EEG data collection. The scalp EEG was acquired in a quiet, relaxed state in a semi-isolated room.

### EEG Data Acquisition

For both patients and controls, video EEG was recorded from a nineteen-channel analog recorder (GALIEO EB Neuro with a camera) placed according to the 10–20 system for 2 h. EEG and video data were recorded in a time-locked pattern. The impedances of the electrodes were kept at <10 kΩ. The sample rate was 256 Hz for all the recordings.

At the EEG monitoring start, the participants were told to keep awake, remain relaxed, keep their eyes closed and not to think of anything in particular for resting-state EEG data acquisition. Then the EEG-video recording lasted for up to 2 h. Following the EEG recording, the EEG patterns were evaluated by two reviewers. Reference montages and average montages were used as well as bipolar montages to assess epileptiform discharges and epileptic seizures. Those patients with frequent typical generalized ca. 3-Hz spike-and-wave complexes were included. The absence seizures were defined as generalized ca. 3-Hz spike-and-wave complexes discharges lasting more than 5 and <15 s. According to previous study (15), the absence onset and end were defined as consistent with onset and end of generalized ca. 3-Hz spike and wave discharges. Only when the two reviewers reached an agreement, the data would be brought into the final analysis.

### EEG Data Preprocessing

The original EEG data were exported in European Data Format (EDF) format. The EEG was bandpass filtered between 1 and 45 Hz with a Hamming window after the FIR filter. Then the electromyogram artifacts and muscle artifacts were completely automated removed using blind source separation. Both electrooculogram artifact and muscle artifact corrections were carried out using a fast ICA algorithm. These procedures were done using an automated EEGlab plugin-in AAR (http://germangh.com). Those EEG matrices with low fractal dimensions would be recognized as artifact components and be removed automatically. Then, the EEG was recomputed to the common average reference. After preprocessing, the EEG of interest was cut out and translated to text format for further ICA analysis. For each subject in AE patients, we extracted five 10 s epoch EEG data for inter-ictal, pre-ictal (pre-seizure), and post-ictal (post-seizure) states. Five 10 s epoch EEG data in the resting state with eyes closed and for each subject in controls. inter-ictal EEG was defined as EEG epochs with eyes closed standing in the time points that there was no seizures onset before and after it within 1 min. Data preprocessing was carried out using MATLAB (R2014a, MathWorks, Inc.) toolbox EEGlab (16).

### Functional ICA

To analyze the functional network divergence of brain activity between different states, an exact Low-Resolution Electromagnetic Tomography (eLORETA) functional ICA method (eLORETA-ICA) was employed. eLORETA is a widely used method to localize multiple distributed cortical sources of EEG data in three-dimensional space (17). The LORETA method has been previously validated in many real human EEG data. The present used eLORETA (V20190617) is an improvement over earlier related version of LORETA (17) or Standardized LORETA (sLORETA) (18). ICA is a mathematical decomposition algorithm that separates the EEG signal into a set of statistically independent components. The technical details of functional ICA has been described in previous studies (19, 20).

In short, the EEG signals of each subject were transformed to cross-spectral EEG matrix for each frequency band using the discrete Fourier transform. Then, these transformed files were used for computing the spectral density for each cortical gray voxel (6,239 voxels) and for each frequency band. Thus, we obtained six eLORETA images of cortical spectral density five frequency bands (delta band: 1–4 Hz; theta band: 4–8 Hz; alpha band: 8–13 Hz; beta band: 13–30 Hz; gamma band: 30–45 Hz.). These data correspond to a “function” of space (cortical voxel) and frequency. After that, the data from each subject in two groups is concatenated. Thus, a matrix including two dimensions were created, in which one dimension corresponds to different subjects, and the other dimension corresponds to space-frequency. This process was known as functional data analysis. When independent component analysis is used to this matrix, general networks are found. This method is defined as functional ICA. Each functional network is composed of five images, one for each frequency band (11). Those components with *Z* scores higher than or equal to “3” were considered statistically significant and selected for later comparisons between two conditions.

In present study, to achieve an optimal number of components. We used sphericity test (21) to compute the eigenvalue of components to estimate the number of components (“9” in this study).

### Statistical Evaluation

According to ICA, we obtained the coefficient of each network component for each subject. One-way ANOVA was used to conduct statistical analysis on the overall difference in coefficients among groups. Only when one variable showed significant differences (*P* < 0.05) in the ANOVA test among the four conditions was the variable considered in further two-by-two comparisons. For group comparisons between AE group and HC group, we used independent sample *t*-test. To compare the network coefficient in different states within the AE group, we used paired *t*-test. The null distribution between two conditions was evaluated by Monte-Carlo simulation with a repetition of 1,000,000 iterations. Only when all tests with *P* < 0.05 were considered statistically significant. A significant difference between two groups or states means they use the same network in a different way.

These steps were carried out using a self-writing MATLAB (R2014a, MathWorks.Inc.) code.

## Results

### Cohort Demographics and Clinical Information

Twenty-one AE patients (aged 9.6 ± 2.9 years) and 21 age- and sex-matched healthy subjects (aged 9.7 ± 2.6 years) were included in the study (Table 1). Artifact-free 1,050-s EEG epochs in inter-ictal, pre-ictal, and post-ictal state EEG for AE patients and in resting state EEG for HC group were obtained, respectively. The AE patients did not take antiepileptic medication at the data acquisition time.

**Table 1:** Demographic information of participants^*^.

	**AE group (*n* = 21)**	**HC group (*n* = 21)**	**χ^2^/*t***	***P***
Sex (male:female)	14:7	13:8	0.10	0.75
Age (years, mean ± SD)	9.6 ± 2.9	9.7 ± 2.6	−0.20	0.84
Medication	No	No	/	/

* *Chi-square test and independent sample t-test were used for sex and age comparisons, respectively*.

### Functional Network Divergences Between AE Patients and HC Subjects

**Figure 1:**
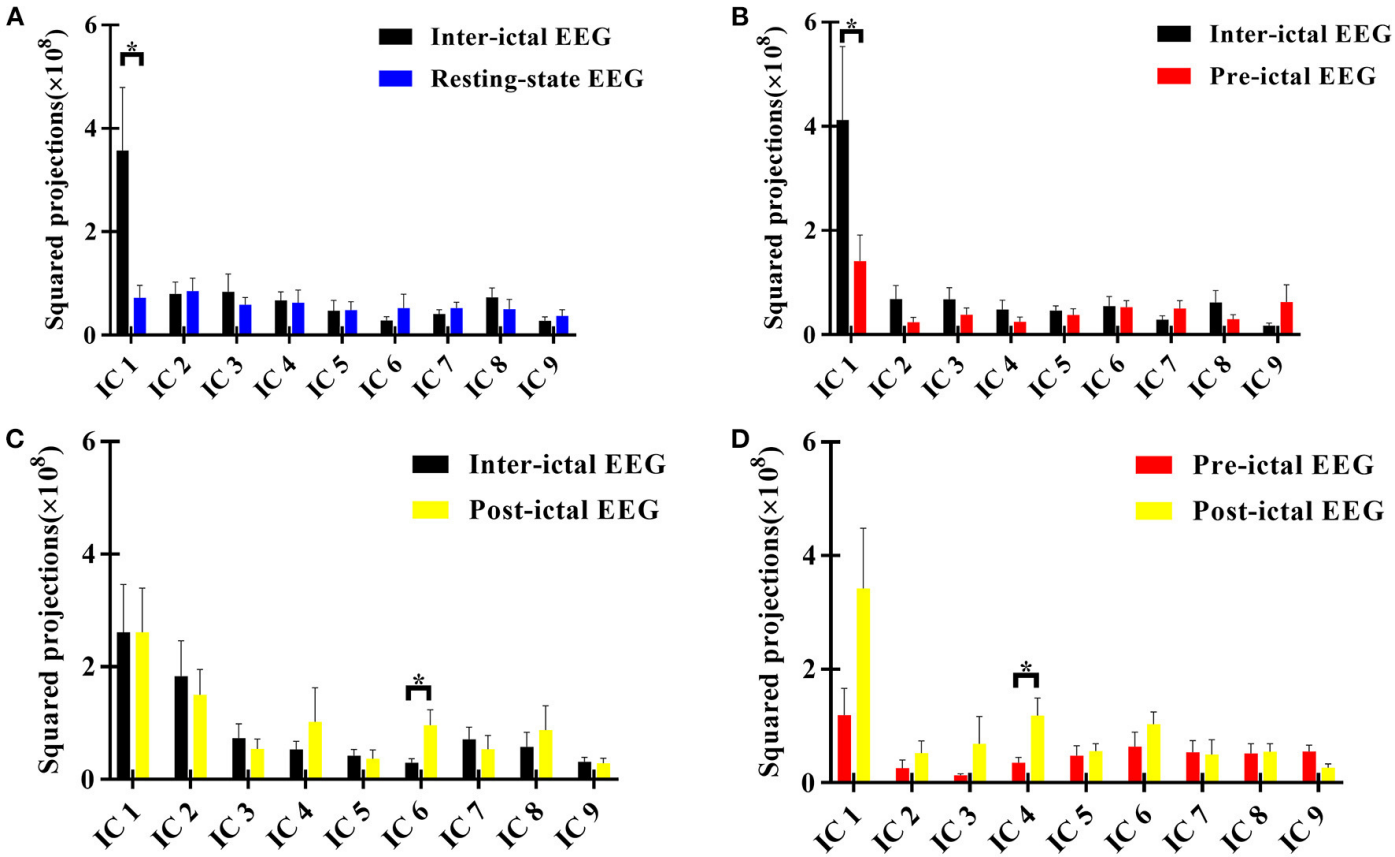
Squared loading comparisons of the components*. **(A)** Inter-ictal EEG compared with the resting-state EEG data of HC group; **(B)** Pre-ictal EEG was compared with inter-ictal EEG within AE group; **(C)** Post-ictal EEG was compared with inter -ictal EEG within AE group; **(D)** Post-ictal EEG compared with pre -ictal EEG within AE group. *P < 0.05, two sample *t-*test between AE group and HC group, and paired *t*-test for different states within AE group. Those components with Z scores lower than 3 were not taken into comparisons. IC, independent component. The null distribution between two conditions was evaluated by Monte-Carlo simulation with a repetition of 1,000,000 iterations (*P* < 0.05).

**Figure 2:**
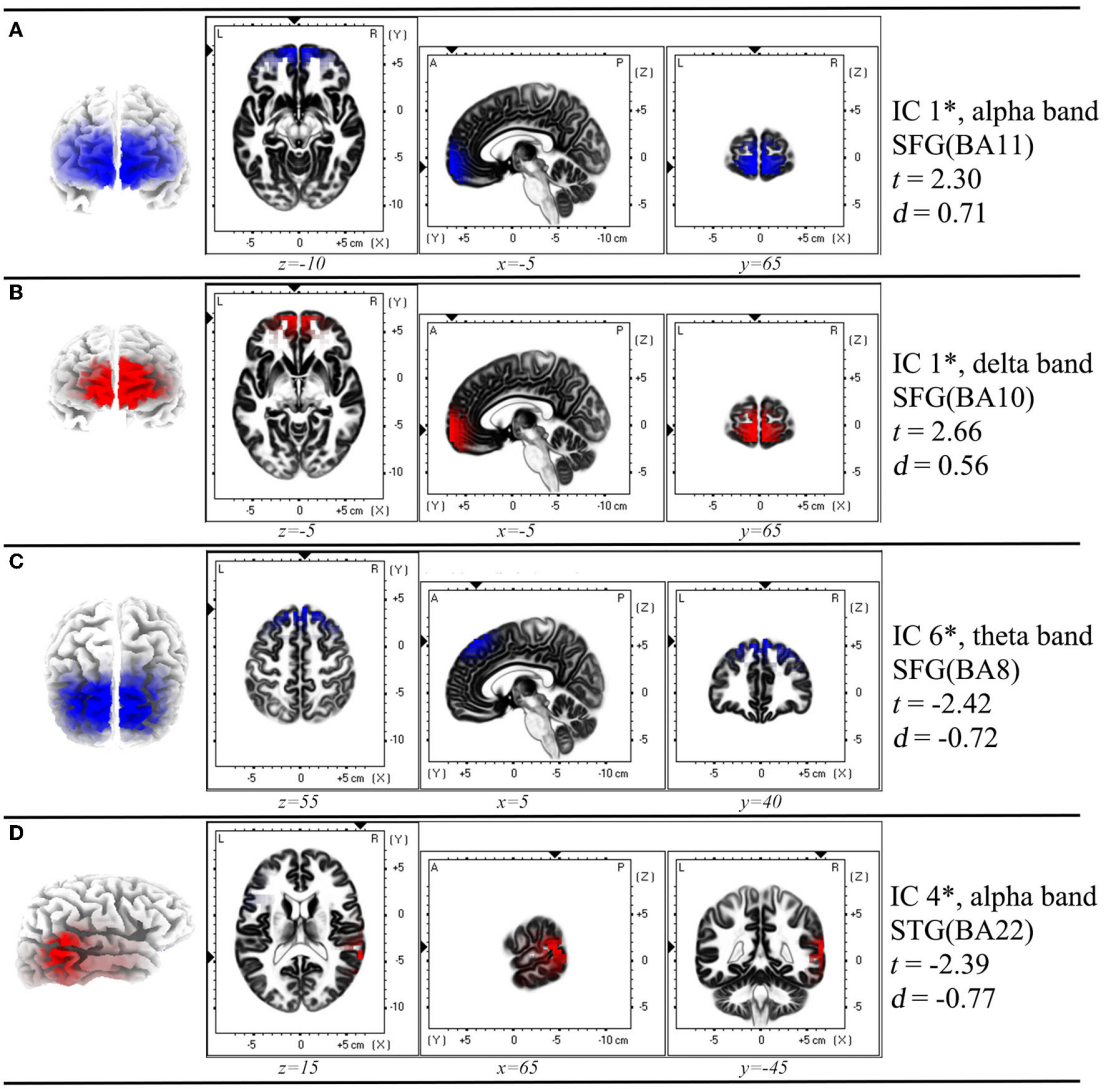
Spatial profile corresponding to the functional independent component that has significant difference in each comparison in Figure 1^*^. **(A)** Inter-ictal EEG compared with the resting-state EEG data of HC group; **(B)** Pre-ictal EEG was compared with inter-ictal EEG within AE group; **(C)** Post-ictal EEG was compared with inter -ictal EEG within AE group; **(D)** Post-ictal EEG compared with pre -ictal EEG within AE group. In each row of figure, the left panel showed the macro location of each IC. In the middle panel, the small triangles along the coordinate axes indicate the maximum electric neuronal activity, and the corresponding peak coordinates are displayed in the lower part of each intercepted image. In the right panel, the IC number, frequency band, location and Cohen's *d* values were presented. ^*^P < 0.05, two sample *t-*test between AE group and HC group, and paired *t*-test for different states within AE group. IC, independent component; SFG, superior frontal gyrus; STG, superior temporal gyrus; BA, Brodmann area; *t, t*-value of *t*-test; *d*, Cohen's *d*-value. The location assignments were shown in MNI space (MNI152-2009c gray matter).

According to the sphericity test, we obtained 9 components in each comparison between two groups or conditions. We found that the most striking differences between the inter-ictal EEG of AE patients and resting-state EEG of HC subjects was in component one which showed that AE patients showed decreased alpha oscillations in regions involving superior frontal gyrus (SFG) (BA11) (Figures 1, 2).

### Functional Network Alteration During Peri-Ictal Periods in AE Patients

It was noted that from the pattern of functional networks changed in different state EEG of AE patients. SFG and superior temporal gyrus (STG) were the key brain regions in the changes of functional network pattern in AE patients. We found that in component one, SFG (BA10) showed maximum decreased brain activity in delta band in pre-ictal state, when comparing with inter-ictal state in AE patients. In addition, compared with pre-ictal state, superior temporal gyrus (STG) (BA 22) in post-ictal state presented maximum increased neural activity in alpha band, which has been displayed in component three. Moreover, from components six, we noted that compared with inter-ictal EEG, post-ictal EEG in AE patients showed significantly decreased theta activity in SFG (BA8) (Figures 1, 2).

## Discussion

Previous reports on AE using MEG/EEG methods typically focused on ca. 3 Hz ictal spike-wave discharges (22, 23). The present study provided a new perspective about the brain network in the inter-ictal stage of AE. We recognized that brain networks changed in inter-ictal state of patients with AE, which was characterized by decreased alpha oscillations. In addition, the pattern of functional networks involving SFG and STG showed significant changes in different states of EEG in AE patients. These findings deepen our understanding of AE from brain electrophysiological aspects.

In clinical practice, normal EEG rhythm often been found in inter-ictal epochs of children with AE, similar to resting-state EEG of healthy controls, which means that it was hard to find potential abnormalities in inter-ictal EEG without spike-wave discharges of AE patients using visual inspection. However, when inter-ictal stage EEG from AE patients was compared with resting-state EEG from healthy controls, there is a significance reduction of alpha activity over SFG (BA11) in AE, which indicated that the altered EEG pattern in AE patients, could not be found by visual inspection, could be obtained by ICA. In addition, the alterations in alpha band in AE patients might suggest that they may possibly showed decreased cognitive function, considering that the modulations of alpha oscillations have been found to be involved in various cognitive domain function such as memory and attention (24–26). In fact, some studies have observed the cognitive deficits in AE (27, 28). In addition, superior frontal gyrus, has been thought to contribute to cognitive function (e.g., memory) (29), showed maximum decreased alpha activity further supported our speculation that AE patients showed potential decreased cognitive function.

From inter-ictal to pre-ictal to post-ictal states, we observed a dynamic evolution of brain activity. Notably, EEG oscillations in SFG showed a prominent issue during the phase transitions, which is in good agreement with earlier functional MRI Studies (30, 31). Although an inherent limitation exists in anatomical accuracy of scalp EEG, this concordance between different techniques performed on independent samples is noteworthy considering that this provides reliable evidences about the import role of SFG in the onset of seizures in AE. Neural regulation technique such as Transcranial Magnetic Stimulation over SFG might be an exciting method in the treatment of seizures (32).

The relationship between absence seizures and the default mode network has been widely detected (33). The SFG, which was the most significant change in our study, is well-known nodes in the default mode network. Thus, our study is in line with previous findings that the default mode network is involved in absence seizures (34). However, the function of default mode network in absence seizures remains enigmatic and widely discussed. The default node network has been confirmed was related to processes of attention and working memory (35) and supports dynamic integration of cognitive and emotional processing (36). some study had confirmed that AE children had abnormal behavior in cognition (4). Thus, we may infer that default mode network in AE had been impaired (37).

It is not unexpected that alpha oscillations in STG increased from pre-ictal to post-ictal, which is consistent with clinical observations that the normal background EEG rhythm will recovery after the terminations of absence episodes in AE. Nevertheless, we noted that frequency bands with maximum alterations from inter-ictal to pre-ictal, from post-ictal to inter-ictal were decreased delta oscillations, and increased theta activity, respectively. One reasonable explanation for these findings was that the cortico-thalamo-cortical network contribute to the onset and termination of seizure in AE (38–40). The slow rhythm (e.g., delta) is usual attributed to the oscillatory of brain cortex (41), while the faster rhythm (e.g., alpha) usually owes to thalamic modulation (42). Therefore, the decreased delta oscillations from inter-ictal to pre-ictal might be caused by the decreased activity of cortex involving SFG. The enhanced alpha activity from pre-ictal to post-ictal might be induced by the recovery of thalamo-cortical loops. The theta activity of SFG in inter-ictal state was stronger than that in post-ictal state might indicate the recovery process of memory because of the close associations of theta waves with memory encoding and retrieval (43, 44). From pre-ictal to post-ictal, the upgraded alpha rhythm may suggest the increased connectivity of cortico-thalamo-cortical network.

Over the past years, EEG dynamics in the pre-seizure state have been widely studied for predicting epileptic seizures (45–47). Many prediction algorithms have been applied to predict the occurrences of seizures, including phase synchronization (48), entropy (49), and etc. However, most studies investigated seizures on mathematical approaches and their results were hard to be clarified with clinical practice. The source localization method used in the present study could provide the rough locations of abnormal brain regions during pre-seizure periods, which is biologically meaningful considering that the brain regions could display more important and useful information than pure mathematical parameters in seizure anticipation. In fact, recent studies have realized the deficiency of traditional mathematical parameters and employed more tools such as whole-brain connectivity or a combination of EEG with functional magnetic resonance imaging (fMRI) (50, 51).

The source assignment method used in this study, eLORETA has been identified showing good concordance with resected zone or intracranial EEG in epilepsy patients (52). This method required no prior knowledge and has a full brain gray map estimated but with a relatively low spatial resolution. In addition, the EEG data used in this study had 19 channels. To assess the localization accuracy of eLORETA for EEG with 19 channels. We compared the eLORETA method between 19-channel-EEG and 61-channel-EEG from a same sample. And we found main source assignments of components from 19 channels and from 61 channels are quite similar. Therefore, we compared these different conditions on the same scale using eLORETA -ICA, which are meaningful considering that this study enrolled those AE patients with high frequency seizures and there were few studies available to explore the EEG pattern during the peri-seizure EEG of AE patients.

Study limitations include a relatively small sample. The used source localization method eLORETA may neglect some information from deep cortical and subcortical regions during the peri-seizure periods due to its low space resolution. The activity of cortico-thalamo-cortical network in peri-ictal period was not detailed investigated using combined EEG-fMRI to acquire an accurate location. In future research, we will continuously collect cases and overcome these limitations for a more comprehensive understanding of children AE.

## Conclusion

Our study provides evidences that the differences between inter-ictal EEG of AE patients and resting-state EEGs of healthy controls can be detected by eLORETA. The EEG networks of epileptic patients before and after absence seizures are significantly changed, which may involve the cortico-thalamo-cortical circuits. Overall, our study has advanced our knowledge about the dynamic process of brain networks for peri-ictal states in AE patients, which provide a better understanding of the onset and termination mechanisms of seizures from electrophysiological view.

## Data Availability Statement

The raw data supporting the conclusions of this article will be made available by the authors, without undue reservation.

## Ethics Statement

The studies involving human participants were reviewed and approved by the Medical Ethics Committee of the Affiliated Hospital of Southwest Medical University. Written informed consent to participate in this study was provided by the participants' legal guardian/next of kin.

## Author Contributions

All authors made a significant contribution to the work reported, whether that is in the conception, study design, execution, acquisition of data, analysis and interpretation, or in all these areas, took part in drafting, revising or critically reviewing the article, gave final approval of the version to be published, have agreed on the journal to which the article has been submitted, and agree to be accountable for all aspects of the work.

## Funding

This work was supported by the Youth Program of the National Natural Science Foundation of China (81804198) and Youth Fund of Southwest Medical University (2018-ZRQN-003).We thank the Special Training Program for Young Scientific and Technological Talents of Southwest Medical University 2020-2022 for sponsorship of JR.

## Conflict of Interest

The authors declare that the research was conducted in the absence of any commercial or financial relationships that could be construed as a potential conflict of interest.

## Publisher's Note

All claims expressed in this article are solely those of the authors and do not necessarily represent those of their affiliated organizations, or those of the publisher, the editors and the reviewers. Any product that may be evaluated in this article, or claim that may be made by its manufacturer, is not guaranteed or endorsed by the publisher.

## Abbreviations

EEG: electroencephalogram
eLORETA: exact lower solution brain electromagnetic tomography
AE: absence epilepsy
BA: Brodmann area
HC: healthy control
SFG: superior frontal gyrus
STG: superior temporal gyrus
ICA: independent component analysis.

## References

[1] Berg AT Shinnar S Levy SR Testa FM Smith-Rapaport S Beckerman B. How well can epilepsy syndromes be identified at diagnosis? A reassessment 2 years after initial diagnosis. Epilepsia. (2000) 41:1269–75. 10.1111/j.1528-1157.2000.tb04604.x 11051121

[2] Jallon P Loiseau P Loiseau J. Newly diagnosed unprovoked epileptic seizures: presentation at diagnosis in CAROLE study Coordination Active du Réseau Observatoire Longitudinal de l'. Epilepsie Epilepsia. (2001) 42:464–75. 10.1046/j.1528-1157.2001.31400.x 11440341

[3] Guo JN Kim R Chen Y Negishi M Jhun S Weiss S. Impaired consciousness in patients with absence seizures investigated by functional MRI, EEG, and behavioural measures: a cross-sectional study. Lancet Neurol. (2016) 15:1336–45. 10.1016/S1474-4422(16)30295-2 27839650 PMC5504428

[4] Caplan R Siddarth P Stahl L Lanphier E Vona P Gurbani S. Childhood absence epilepsy: behavioral, cognitive, and linguistic comorbidities. Epilepsia. (2008) 49:1838–46. 10.1111/j.1528-1167.2008.01680.x 18557780

[5] Henkin Y Sadeh M Kivity S Shabtai E Kishon-Rabin L Gadoth N. Cognitive function in idiopathic generalized epilepsy of childhood. Dev Med Child Neurol. (2005) 47:126–32. 10.1017/S0012162205000228 15707236

[6] Bai X Vestal M Berman R Negishi M Spann M Vega C. Dynamic time course of typical childhood absence seizures: EEG, behavior, and functional magnetic resonance imaging. J Neurosci. (2010) 30:5884–93. 10.1523/JNEUROSCI.5101-09.2010 20427649 PMC2946206

[7] Carney PW Masterton RAJ Harvey AS Scheffer IE Berkovic SF Jackson GD. The core network in absence epilepsy Differences in cortical and thalamic BOLD response. Neurology. (2010) 75:904–11. 10.1212/WNL.0b013e3181f11c06 20702791

[8] Tenney JR Kadis DS Agler W Rozhkov L Altaye M Xiang J. Ictal connectivity in childhood absence epilepsy: associations with outcome. Epilepsia. (2018) 59:971–81. 10.1111/epi.14067 29633248

[9] Liu H Tang H Wei W Wang G Du Y Ruan J. Altered peri-seizure EEG microstate dynamics in patients with absence epilepsy. Seizure. (2021) 88:15–21. 10.1016/j.seizure.2021.03.020 33799135

[10] Ventouras EM Ktonas PY Tsekou H Paparrigopoulos T Kalatzis I Soldatos CR. Independent component analysis for source localization of EEG sleep spindle components. Comput Intell Neurosci. (2010) 2010:329436. 10.1155/2010/329436 20369057 PMC2847376

[11] Aoki Y Ishii R Pascual-Marqui RD Canuet L Ikeda S Hata M. Detection of EEG-resting state independent networks by eLORETA-ICA method. Front Hum Neurosci. (2015) 9:e00031. 10.3389/fnhum.2015.00031 25713521 PMC4322703

[12] Xiao F Lu C Zhao D Zou Q Xu L Li J. Independent component analysis and graph theoretical analysis in patients with narcolepsy. Neurosci Bull. (2019) 35:743–55. 10.1007/s12264-018-0307-6 30421271 PMC6616568

[13] Canuet L Ishii R Pascual-Marqui RD Iwase M Kurimoto R Aoki Y. Resting-state EEG source localization and functional connectivity in schizophrenia-like psychosis of epilepsy. PLoS ONE. (2011) 6:e27863. 10.1371/journal.pone.0027863 22125634 PMC3220705

[14] Engel J Jr. International League Against Epilepsy. A proposed diagnostic scheme for people with epileptic seizures and with epilepsy: report of the ILAE Task Force on Classification and Terminology. Epilepsia. (2001) 42:796–803. 10.1046/j.1528-1157.2001.10401.x 11422340

[15] Moeller F LeVan P Muhle H Stephani U Dubeau F Siniatchkin. Absence seizures: individual patterns revealed by EEG-fMRI. Epilepsia. (2010) 51:2000–10. 10.1111/j.1528-1167.2010.02698.x 20726875 PMC3769289

[16] Delorme A Makeig S. EEGLAB: an open source toolbox for analysis of single-trial EEG dynamics including independent component analysis. J Neurosci Methods. (2004) 134:9–21. 10.1016/j.jneumeth.2003.10.009 15102499

[17] Pascual-Marqui RD Michel CM Lehmann D. Low resolution electromagnetic tomography: a new method for localizing electrical activity in the brain. Int J Psychophysiol. (1994) 18:49–65. 10.1016/0167-8760(84)90014-X 7876038

[18] Pascual-Marqui RD. Standardized low-resolution brain electromagnetic tomography (sLORETA): technical details. Methods Find Exp Clin Pharmacol. (2002) 24(Suppl. D):5–12. 12575463

[19] Pascual-Marqui RD Biscay-Lirio RJ. Interaction patterns of brain activity across space, time and frequency. Part I: methods. arXiv [Preprint]. (2011) arXiv:1103.2852. 10.48550/arXiv.1103.2852

[20] Pascual-Marqui R Kochi K Lehmann D Koukkou M Kinoshita T. Functional independent components: Revealing cortico-cortical, cross-frequency interactions. Jpn J Pharmacol EEG. (2011) 12:53–8. 10.5167/uzh-48682

[21] Bartlett MS. A note on the multiplying factors for various χ^2^ approximations. J Royal Stat Soc Series B (Methodological). (1954) 16:296–8. 10.1111/j.2517-6161.1954.tb00174.x

[22] Tenney JR Fujiwara H Horn PS Jacobson SE Glauser TA Rose DF. Focal corticothalamic sources during generalized absence seizures: a MEG study. Epilepsy Res. (2013) 106:113–22. 10.1016/j.eplepsyres.2013.05.006 23764296

[23] Amor F Baillet S Navarro V Adam C Martinerie J Quyen MLV. Cortical local and long-range synchronization interplay in human absence seizure initiation. Neuroimage. (2009) 45:950–62. 10.1016/j.neuroimage.2008.12.011 19150654

[24] Wianda E Ross B. The roles of alpha oscillation in working memory retention. Brain Behav. (2019) 9:e01263. 10.1002/brb3.1263 30887701 PMC6456781

[25] Klimesch W. Alpha-band oscillations, attention, and controlled access to stored information. Trends Cogn Sci. (2012) 16:606–17. 10.1016/j.tics.2012.10.007 23141428 PMC3507158

[26] Foxe J Snyder A. The role of alpha-band brain oscillations as a sensory suppression mechanism during selective attention. Front Psychol. (2011) 2:e00154. 10.3389/fpsyg.2011.00154 21779269 PMC3132683

[27] Masur D Shinnar S Cnaan A Shinnar RC Clark P Wang J. Pretreatment cognitive deficits and treatment effects on attention in childhood absence epilepsy. Neurology. (2013) 81:1572–80. 10.1212/WNL.0b013e3182a9f3ca 24089388 PMC3806916

[28] Maheshwari A Akbar A Wang M Marks RL Yu K Park S. Persistent aberrant cortical phase-amplitude coupling following seizure treatment in absence epilepsy models. J Physiol. (2017) 595:7249–60. 10.1113/JP274696 28901011 PMC5709336

[29] du Boisgueheneuc F Levy R Volle E Seassau M Duffau H Kinkingnehun S. Functions of the left superior frontal gyrus in humans: a lesion study. Brain. (2006) 129:3315–28. 10.1093/brain/awl244 16984899

[30] Xu CP Zhang SW Fang T Manxiu M Chencan Q Huafu C. Altered functional connectivity within and between brain modules in absence epilepsy: a resting-state functional magnetic resonance imaging study. Biomed Res Int. (2013) 2013:734893. 10.1155/2013/734893 24191250 PMC3804038

[31] Luo C Li Q Lai Y Xia Y Qin Y Liao W. Altered functional connectivity in default mode network in absence epilepsy: a resting-state fMRI study. Hum Brain Mapp. (2011) 32:438–49. 10.1002/hbm.21034 21319269 PMC6870112

[32] Pereira LS Muller VT da Mota Gomes M Rotenberg A Fregni F. Safety of repetitive transcranial magnetic stimulation in patients with epilepsy: a systematic review. Epilepsy Behav. (2016) 57:167–76. 10.1016/j.yebeh.2016.01.015 26970993

[33] Sakurai K Takeda Y Tanaka N Kurita T Shiraishi H Takeuchi F. Generalized spike-wave discharges involve a default mode network in patients with juvenile absence epilepsy: a MEG study. Epilepsy Res. (2010) 89:176–84. 10.1016/j.eplepsyres.2009.12.004 20061122

[34] Miao A Tang L Xiang J Guan Q Ge H Liu H. Dynamic magnetic source imaging of absence seizure initialization and propagation: a magnetoencephalography study. Epilepsy Res. (2014) 108:468–80. 10.1016/j.eplepsyres.2014.01.006 24534760

[35] Owen AM McMillan KM Laird AR Bullmore E. N-back working memory paradigm: a meta-analysis of normative functional neuroimaging studies. Hum Brain Mapp. (2005) 25:46–59. 10.1002/hbm.20131 15846822 PMC6871745

[36] Raichle ME Mintun MA. Brain work and brain imaging. Ann Rev Neurosci. (2006) 29:449–76. 10.1146/annurev.neuro.29.051605.112819 16776593

[37] Danielson NB Guo JN Blumenfeld H. The default mode network and altered consciousness in epilepsy. Behav Neurol. (2011) 24:55–65. 10.1155/2011/912720 21447899 PMC3150226

[38] Maheshwari A Noebels JL. Monogenic models of absence epilepsy: windows into the complex balance between inhibition and excitation in thalamocortical microcircuits. Prog Brain Res. (2014) 213:223–52. 10.1016/B978-0-444-63326-2.00012-0 25194492

[39] Evangelista E Bénar C Bonini F Carron R Colombet B Régis. Does the thalamo-cortical synchrony play a role in seizure termination? Front Neurol. (2015) 6:e00192. 10.3389/fneur.2015.00192 26388834 PMC4555023

[40] Shepherd GMG Yamawaki N. Untangling the cortico-thalamo-cortical loop: cellular pieces of a knotty circuit puzzle. Nat Rev Neurosci. (2021) 22:389–406. 10.1038/s41583-021-00459-3 33958775 PMC9006917

[41] Csercsa R Dombovari B Fabo D Wittner L Eross L Entz L. Laminar analysis of slow wave activity in humans. Brain. (2010) 133:2814–29. 10.1093/brain/awq169 20656697 PMC3105490

[42] Hughes SW Crunelli V. Thalamic mechanisms of EEG alpha rhythms and their pathological implications. Neuroscientist. (2005) 11:357–72. 10.1177/1073858405277450 16061522

[43] Herrmann CS Strüber D Helfrich RF Engel AK. EEG oscillations: from correlation to causality. Int J Psychophysiol. (2016) 103:12–21. 10.1016/j.ijpsycho.2015.02.003 25659527

[44] Klimesch W. EEG alpha and theta oscillations reflect cognitive and memory performance: a review and analysis. Brain Res Brain Res Rev. (1999) 29:169–95. 10.1016/S0165-0173(98)00056-3 10209231

[45] Navarro V Martinerie J Le Van Quyen M Clemenceau S Adam C Baulac. Seizure anticipation in human neocortical partial epilepsy. Brain. (2002) 125:640–55. 10.1093/brain/awf048 11872619

[46] Navarro V Martinerie J Le Van Quyen M Baulac M Dubeau F Gotman J. Seizure anticipation: do mathematical measures correlate with video-EEG evaluation? Epilepsia. (2005) 46:385–96. 10.1111/j.0013-9580.2005.15504.x 15730536

[47] Shoeibi A Khodatars M Ghassemi N Jafari M Moridian P Alizadehsani R. Epileptic seizures detection using deep learning techniques: a review. Int J Environ Res Public Health. (2021) 18:5780. 10.3390/ijerph18115780 34072232 PMC8199071

[48] Kuhlmann L Freestone D Lai A Burkitt AN Fuller K Grayden DB. Patient-specific bivariate-synchrony-based seizure prediction for short prediction horizons. Epilepsy Res. (2010) 91:214–31. 10.1016/j.eplepsyres.2010.07.014 20724110

[49] Yang Y Zhou M Niu Y Li C Cao R Wang B. Epileptic seizure prediction based on permutation entropy. Front Comput Neurosci. (2018) 12:e00055. 10.3389/fncom.2018.00055 30072886 PMC6060283

[50] Youssofzadeh V Agler W Tenney JR Kadis DS. Whole-brain MEG connectivity-based analyses reveals critical hubs in childhood absence epilepsy. Epilepsy Res. (2018) 145:102–9. 10.1016/j.eplepsyres.2018.06.001 29936300

[51] Tenney JR Williamson BJ Kadis DS. Cross-frequency coupling in childhood absence epilepsy. Brain Connect (2021). 10.1089/brain.2021.0119. [Epub ahead of print]. 34405685

[52] van Mierlo P Vorderwulbecke BJ Staljanssens W Seeck M Vulliemoz S. Ictal EEG source localization in focal epilepsy: review and future perspectives. Clin Neurophysiol. (2020) 131:2600–16. 10.1016/j.clinph.2020.08.001 32927216

